# The Laboratory Automation Protocol (LAP) Format and Repository: a platform for enhancing workflow efficiency in synthetic biology

**DOI:** 10.1021/acssynbio.3c00397

**Published:** 2023-11-20

**Authors:** Ana-Mariya Anhel, Lorea Alejaldre, Ángel Goñi-Moreno

**Affiliations:** 1 Centro de Biotecnología y Genómica de Plantas, Universidad Politécnica de Madrid (UPM)-Instituto Nacional de Investigatión y Tecnología Agraria y Alimentaria (INIA/CSIC), 28223, Madrid, Spain

**Keywords:** Protocol, Automation, Standard, Repository

## Abstract

Laboratory automation deals with eliminating manual tasks in high-throughput protocols. It therefore plays a crucial role in allowing fast and reliable synthetic biology. However, implementing open-source automation solutions often demands experimental scientists to possess scripting skills, and even when they do, there is no standardized toolkit available for their use. To address this, we present the Laboratory Automation Protocol (LAP) Format and Repository. LAPs adhere to a standardized script-based format, enhancing end-user implementation and simplifying further development. With a modular design, LAPs can be seamlessly combined to create customized, target-specific workflows. Furthermore, all LAPs undergo experimental validation, ensuring their reliability. Detailed information is provided within each repository entry, allowing users to validate LAPs in their own laboratory settings. We advocate for the adoption of the LAP Format and Repository as a community resource, which will continue to expand, improving the reliability and reproducibility of automation processes.

Laboratories have been striving to “reduce the manual effort in repetitive tasks as much as possible”^1^ for decades now. The objective is not only to increase production rates but also to replace error-prone manual steps with robust and reliable processes, ultimately enhancing reproducibility.^2^ The degree to which this goal has been accomplished varies depending on the nature of the scientific activity. Industrial and clinical laboratories, for example, were among the earliest adopters of automation due to their requirements for reproducibility, scalability, managing large sample volumes, and high-throughput screening.^3^ In contrast, fundamental research laboratories have been slower to implement automation processes,^4^ and several factors may contribute to this. Firstly, the nature of problems encountered in basic research labs does not always necessitate automation processes, as a significant portion of daily activities involves result observation and hypothesis generation—without production pressures. Secondly, molecular biology and microbiology laboratories often lack formal interdisciplinary training, particularly in the fields of robotics and automation.^5^ Last, academic laboratories often lack the financial resources to access automation equipment.

The advent of synthetic biology^6^ has transformed this situation. The rational engineering of predetermined functions in living systems not only requires interdisciplinary collaboration among biological sciences, computation, physics, and data science, but also entails automation throughout the design-build-test-learn (DBTL) life-cycle.^7,8^ Precision and accuracy have, to some extent, superseded scalability as primary objectives, particularly in basic research laboratories. And the availability of open-source automation equipments has facilitated access to such applications. Even relatively small tasks, such as constructing and characterising a plasmid vector, can now be effectively carried out using automation facilities.^9^ Numerous tools and methods have emerged in the past decade to automate the design of genetic constructs^10,11^ and establish end-to-end workflows.^12^ These workflows involve collaboration among various disciplines, encompassing design, mathematical modelling, genetic engineering, and result analysis, all working towards a shared objective. Currently, there is significant room for improvement at the convergence of software, hardware, and wetware,^13^ which paves the way for laboratory automation in synthetic biology laboratories, assisting in addressing future challenges.^14–16^

At the heart of open-source automation is the utilization of program scripts, which are sets of instructions that encode molecular biology protocols. These scripts serve as inputs for liquid handling robots to automate the execution of these protocols. In this context, we introduce a resource called the Laboratory Automation Protocol (LAP) Format and Repository. Its purpose is to formalize and store experimentally validated automation scripts by providing set of standardized guidelines to accelerate script development and facilitate implementation by the end-user. It is important to note that our intention is not to replace previous initiatives but rather to complement them. For example, recent efforts in protocol-specific language description^17,18^ can be used to speed up the process of defining protocol primitives. We suggest to leverage all efforts by providing a standardized structure, known as the LAP format, to improve compatibility and facilitate development. Additionally, we offer a repository to facilitate the sharing of LAP scripts and foster collaboration among researchers. This follows a similar approach employed in other stages of the DBTL (Design-Build-Test-Learn) life cycle. For instance, in the design stage, relevant information derived from genetic construct designs, such as DNA sequences or regulatory interactions, can be efficiently captured using the SBOL^19,20^ (Synthetic Biology Open Language) standard and stored in dedicated repositories like SynBioHub.^21^ Another illustration is the utilization of the SEVA^22^ (Standard European Vector Architecture) format and plasmid vectors during the build stage.

Figure 1 illustrates the features of the LAP Format, a standardized scripting frame-work. Adopting a standard format not only accelerates the creation of new protocols but also streamlines the implementation of high-throughput workflows that involve multiple sequential protocols. The proposed format entails a structured approach wherein customize variables for each entry are separated into another file, allowing users to tailor the protocol without access to the script. Given the availability of various software tools designed to generate scripts based on user specifications,^23–27^ we strongly advocate for adhering to standard guidelines for script structure to promote collaboration in automated protocol development.

All protocols listed in the LAP Repository conform to this format, and any new additions must also adhere to it going forward. The file naming convention (Figure 1A) follows a specific structure: repository name (LAP), a concise description of the protocol’s functionality (e.g., ColonyCounterSelection), the platform used (e.g., the OT2 liquid handling robot), and the version number (e.g., 1.0.0), separated by hyphens. For example, a protocol file could be named LAP-ColonyCounterSelection-OT2-1.0.0.

Each LAP file is divided into three sections: Classes, Functions, and Body (Figure 1B). User interaction with the protocol occurs through the Classes section, where variables specific to the protocol are defined. While values may vary, the variable names remain consistent across protocols, ensuring standardization and easier understanding of the protocol. This interaction is facilitated by a “Customizable Variable File” that users fill in with information about the variables and corresponding values. End-users only interact with this variable file and don’t need to have any scripting background to implement open-source automation in their laboratories. The Customizable Variable File can be in various formats such as xlsx, csv, json, txt, etc. The Classes section of the LAP utilizes this file to complete the protocol specifications. The Functions section contains standardized functions that can be easily copied and pasted between different scripts, depending on the requirements of the specific protocol. Users interested in developing new LAPs will likely find these functions valuable. All functions are located independently in the SetFunctions directory of the LAP GitHub repository. Any new functions developed for future LAP entries will also be added to this same folder. The Body section is where the actual protocol is coded, and its content is highly specific to each script e.g., liquid transferring. Developers (distinct from end users) can edit existing LAPs and create new ones by modifying the Body section according to their requirements. Structuring the script in this way allows for easier protocol customization, execution and re-use of the different code blocks.

LAPs are developed with a modular approach (Figure 1C), which is crucial as it allows for the seamless combination of multiple LAPs in a sequential manner to execute more complex protocols, such as workflows. For example, the LAP protocol *Cell Inoculation in Different Media* can be used as part of a workflow to genotype bacterial colonies along with LAP protocol *2 criteria Counter Selection* and *PCR Mix Preparation and Temperature Profile* as described in https://dx.doi.org/10.17504/protocols.io.kqdg394jzg25/v1. LAP protocols can be re-used within different workflows. Furthermore, this modularity is tailored to the end-user as it facilitates the sharing of protocol files among different laboratories and reduces redundancy when creating new workflows.

The LAP Repository (Figure 2) is accessible through the web at www.laprepo.com. The landing page (Figure 2A) serves as an introduction to LAP and highlights our perspective on automation, emphasizing key features such as modularity and standardization. The repository is organized into four sub-pages: *LAP Format*, which provides an overview of the LAP Format as described in Figure 1; *Repository*, where users can explore the currently available protocols listed in the repository; *Further info*, which describes additional advantages of the LAP scripts, including the open-source nature of the project; and *Contact*, that provides contact details for the LAP Repository. Importantly, under *Further info* there is a video explanation summarizing how users can contribute to this effort by providing LAP scripts that comply with the LAP Format, along with comprehensive descriptions of their functionality and the specific variables used for achieving the desired results. LAP curators will carefully review the submitted data before uploading it to the repository.

In the Repository sub-page, which displays the list of protocols (Figure 2B), users can either browse through the available LAPs or search according to their name or function. Currently, the repository includes protocols for various purposes such as modular cloning (MoClo) assembly, sample consolidation in a single 96-well plate, 2-criteria counter selection, cell inoculation in different media, PCR mix preparation and temperature profile. These protocols are designed to be versatile and can be applied to a wide range of experiments and workflows. To date, the LAP repository does not include the description of workflows; however, several LAPs have been used to build an example workflow available at https://dx.doi.org/10.17504/protocols.io.kqdg394jzg25/v1.

Additionally, the Repository sub-page features LAP Utilities (LAPu). LAP Utilities is a section that encompasses protocols or scripts that may not strictly adhere to the LAP format, such as those that do not require a variables file input. However, these utilities play a significant role in the high-throughput dimension of automation and are utilized within workflows. Currently, there is one LAPu available for alignment and annotation using BLASTn, which is specifically used for annotating sequencing results from samples screened through an automation workflow, as showcased in https://dx.doi.org/10.17504/protocols.io.kqdg394jzg25/v1.

Moving forward, all future protocols and utilities will be directly added to this list. Furthermore, new additions are not limited to a specific platform or liquid-handling robot. While the protocols listed were developed using the same platform employed in our laboratory, we strongly encourage researchers to submit protocols for other platforms as well. Also, this includes diverse methods like imaging^28^ and colony picking.^29^

Users can access detailed information for each LAP by clicking on the *More Info* or the *Download* button. The *More Info* option presents the information in a dedicated online sub-page, while the *Download* option allows users to obtain the information in a document format. LAP scripts are downloaded along with a PDF file providing instructions to run the script, an input file that that corresponds to the validation described for each protocol and a YAML file with the script’s metadata. The provided information follows a standardized layout, ensuring consistency among LAPs and improving understanding. This format is expected to be followed for any new protocols added to the repository. Along with the LAP files (i.e., the LAP protocol and the Customizable Variable File), users will find a high-level description of the protocol’s objectives, aims, and scope. The validation of the script for a specific use case is also included, along with step-by-step instructions for running the LAP and troubleshooting potential errors. The customizable variable file downloaded with each script includes by default values used for the validation described in the *More Info* sub-page for each protocol. Practical details, such as code versions and packages, the robotic platform used, and other metadata and requirements, are also provided. Each LAP is accompanied by a figure (Figure 3) that visually illustrates the instructions described in the text, enhancing user comprehension.

By synergistically combining the forces of standardization and automation,^30^ remarkable improvements in reproducibility and scalability throughout the DBTL life-cycle can be achieved. The LAP Format and Repository serves as a bridge in this endeavor, providing an open-source platform. This resource establishes a connection with existing and future initiatives in multifaceted ways. For instance, existing (and future) protocol generators are encouraged to embrace the LAP Format, thus boosting protocol sharing and collaboration. Furthermore, the capture and meticulous representation of protocol information^31,32^ can shape the formalization of protocols at an earlier stage, providing baseline tools and abstractions to ease protocol design. Moreover, the standardization efforts focused on genetic parts, exemplified by initiatives such as the SEVA Golden Standard collection^22^ and the SEVA inverter package,^33^ unleash their full potential within automation facilities, positioning automation protocols at the core of scalable and robust synthetic biology.

## Methods

### LAP Interaction with User

Each LAP consists of two essential components. The first part is a program written in the platform’s programming language, responsible for executing the instructions required to accomplish the protocol’s objectives. The second part is a customizable, structured, and human-readable file that contains various variables. Users can modify this *Customizable Variable File* (CVF) to tailor the program according to their specific experimental requirements. This file can be saved in different formats such as JSON, XML, or CSV. The program will parse the variable file, extracting the necessary information and dynamically adapting the protocol accordingly. For the LAPs currently available (in the release version), the program format is Python, while the CVF format is XML.

One example of a Customizable Variable File would be an Excel file with multiple sheets stating the pipettes the liquid handling robot will use, the number of samples we want to handle, volumes of each reactive, and other variables. The instructions on how to fill the variable file are set by the developer and are included in each entry of the repository.

This interaction between the script and the variable file allows the user to personalize their experimental procedures without requiring any programming expertise.

### The LAP Format: Classes

The LAP programs employ different classes with properties and methods to facilitate communication with the script’s body and functions. These classes play a crucial role in parsing the variables provided in the CVF, performing error checking, and generating user-friendly error messages related to possible variable errors, such as trying to transfer to a well more volume than its maximum. An important feature is that these enable accurate calculation of required volumes based on the user’s variables, thus maximising efficiency.

Common classes are shared across different protocols. For instance, the *UserVariables* class is responsible for reading, parsing, and validating the CVF provided by the user. It ensures the values are correctly formatted and checks for potential errors. The *SetParameters* class then takes the object created by *UserVariables* and performs calculations to fill various objects or variables required by the script’s body. This process allows for efficient parameter handling and preparation.

LAPs can include specific classes tailored to unique protocol functionalities. For example, the *MapLabware* class creates a data frame with the dimensions of a given labware, fills it with values such as sample names, and finally exports it to a CSV file that the user can import from the liquid handling robot. This feature is highly beneficial in tracking colony samples throughout an experimental workflow, providing traceability during the experimental procedure.

While the content of these classes may vary depending on the specific variables required for a given protocol, it is encouraged to adopt consistent naming for similar variable concepts. For instance, the right-mounted pipette can always be referred to as *pipR*. This approach facilitates class translation between different LAP programs and enhances code readability and maintenance for developers. In addition, some functions can require objects that have properties with specific names and values to be used.

### The LAP Format: Functions

The LAP protocols encompass a variety of functions essential for executing the script’s core tasks. These functions can be categorized into different types based on their specificity.

Firstly, some functions are widely shared among protocols due to their utility in liquid-handling scripts. For example, the *give_me_optimal_pipette* function that determines the most suitable pipette to minimize the movements required for transferring a specific volume from one location to another. Shared standard functions ensure the codebase’s modularity, reusability and readability.

Secondly, some functions are specific to the individual protocols and tailored to address the unique requirements of a particular experiment. For instance, *distribute_z_tracking_falcon15ml* performs distribution to a specified list of positions while tracking the liquid level to prevent the pipette from getting wet, and it is tailored to be used in falcons of 15mL, so it is not recommended to be used in an experiment that will use a 50mL falcon to hold reagents.

Both types of functions require name consistency inside the previously discussed classes, emphasizing the importance of consistent naming conventions for variables of the same type. An example of such a function is *check_tip_and_pick* which verifies whether a given pipette has a tip attached and, if not, establishes a tip rack so the pipette can pick a tip. For this purpose, the function needs the *UserVariable* class to have a property called *APINameTipR* and *APINameTipL* that will contain the name of the tipracks that the pipettes need.

### The LAP Format: Body

Every LAP protocol has a unique script body that utilizes previously established classes and functions to accomplish specific goals. This personalized method enables each LAP protocol to be customized and adjusted to meet the exact needs of an experiment.

### LAP Utilities

The LAP utilities (LAPu) are intended to streamline the workflow associated with data analysis and provide researchers with efficient tools for processing and extracting information from their high-throughput experimental data. While they may not be specifically tailored for robotic liquid handling operations, they offer valuable functionalities to researchers involved in acquiring and processing large volumes of data.

The different functionalities and platform of usage makes the entries in the LAP Utilities section deviate from the LAP structure and their set of classes and functions.

### Validation

All LAPs have undergone experimental validation. For each protocol, both input and output files from the validation process are provided, serving as a reference for verifying the correct functionality of the LAP. Furthermore, within each LAP, there is a dedicated section called *Validation* that offers additional experimental information. This includes details such as the specific reagents and conditions used during the original validation, ensuring reproducibility. The simulated labware setup and specified reagent quantities are also outlined. Therefore, to validate a LAP in a new laboratory, it is sufficient to utilize the default input files provided, along with the information presented in the validation section.

## Figures and Tables

**Figure 1:**
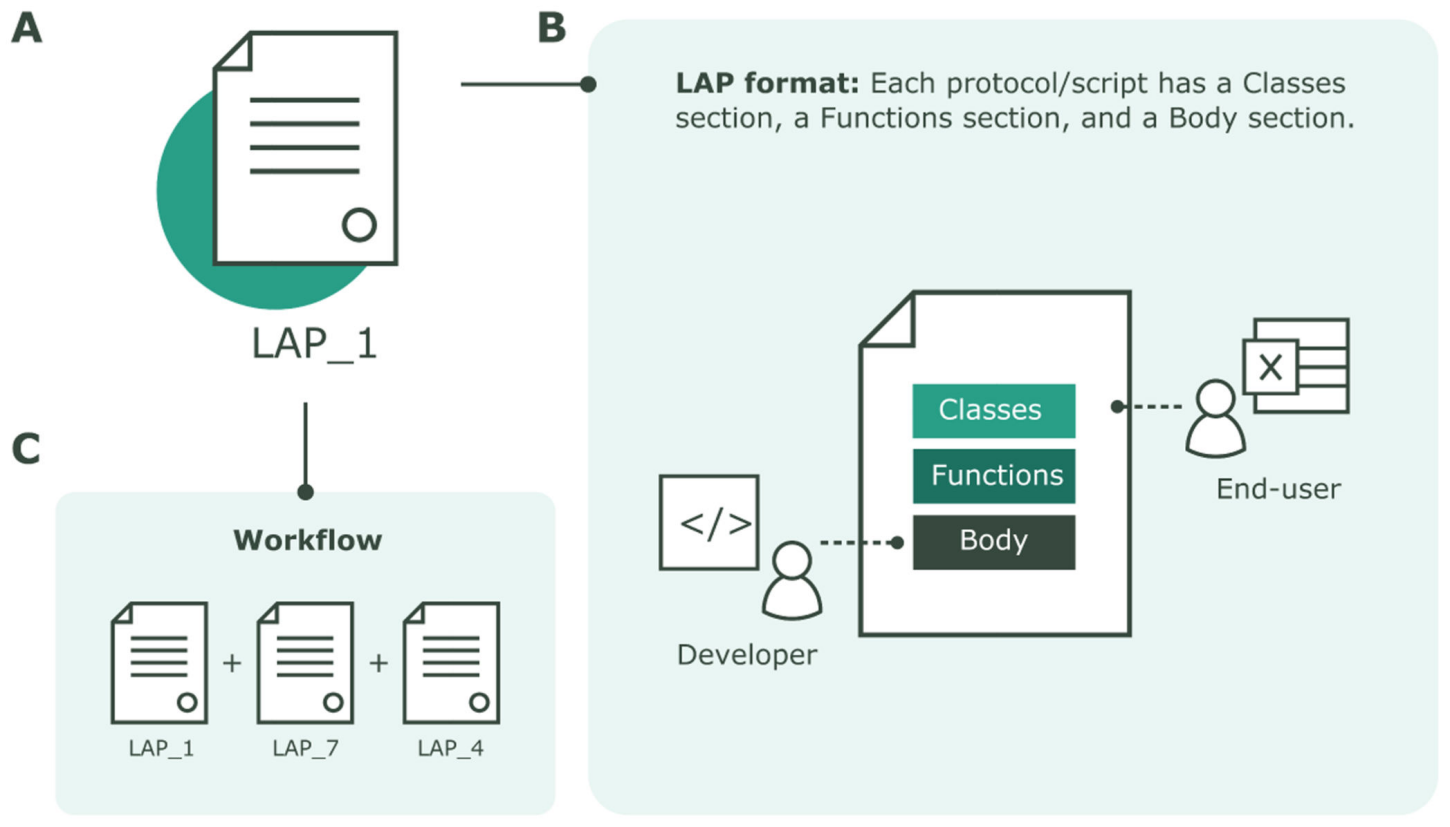
The LAP Format – A standard to build modular and reproducible scripts. Automation scripts are standardized, facilitating the coding of new LAPs with the aim of maximizing reusability and modularity. **A.** The LAP name or identifier should follow this nomenclature: repository name, brief script description, platform used, and version number, separated by hyphens. For example: LAP-ColonyCounterSelection-OT2-1.0.0. **B.** Each LAP consists of three sections: Classes, where users define protocol variables; Functions, which are standardized across all scripts; and Body, where developers can edit and generate protocols. **C.** Modularity. Various LAPs can be sequentially merged in order to run a more complex workflow.

**Figure 2:**
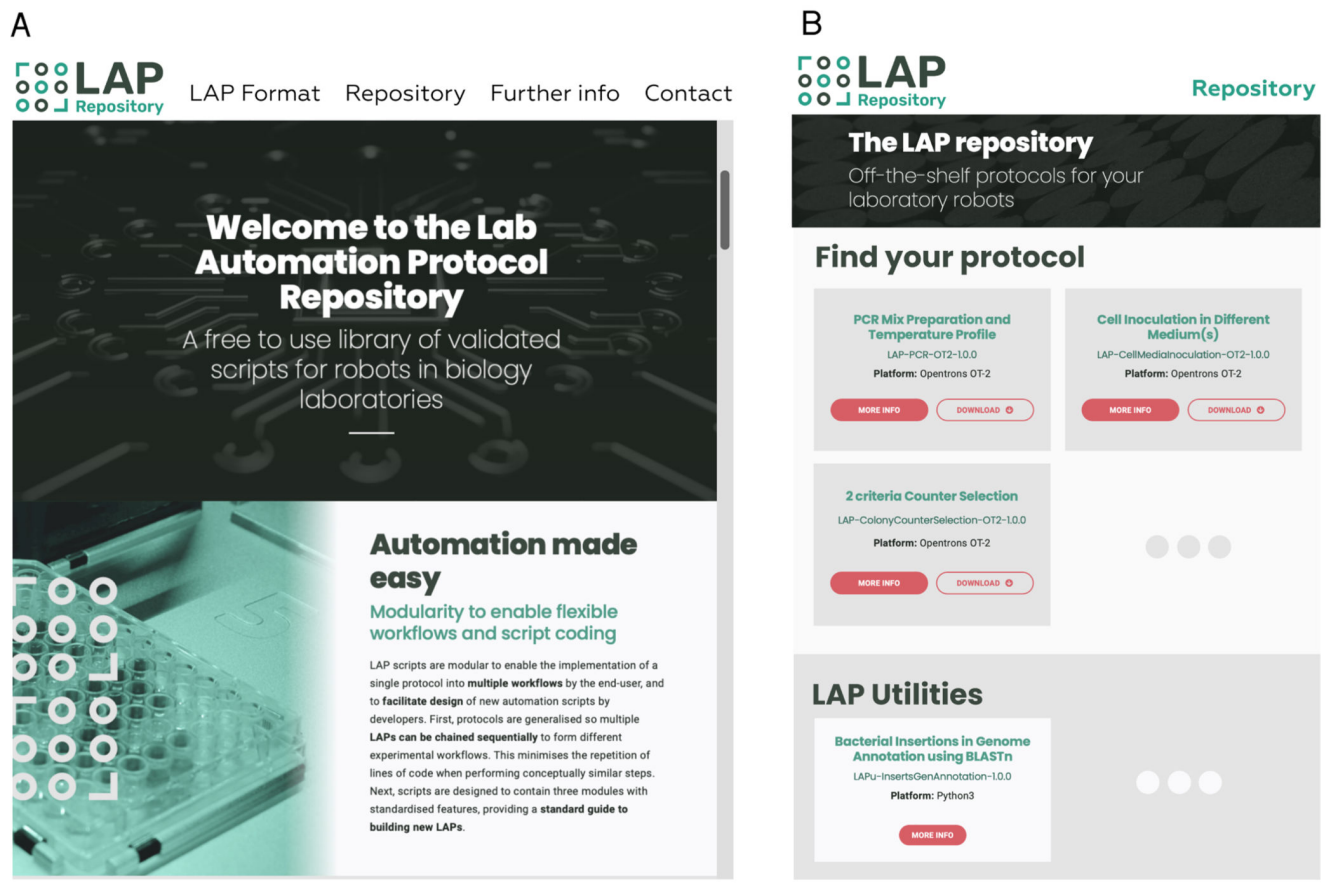
The LAP Repository – www.laprepo.com. Details of the home page and the Repository. **A.** The landing page showcases the repository, providing LAP descriptions and key features. Users can access four subpages: LAP Format, which explains the standard format; Repository, listing available LAPs; Further Info, containing technical details and information on contributing to the repository; and Contact. **B.** The Repository. This page provides the list of available protocols. Users can click on a specific protocol to access a dedicated page with detailed information. Additionally, the page includes a compilation of LAP Utilities, which are valuable tools for working with LAPs.

**Figure 3:**
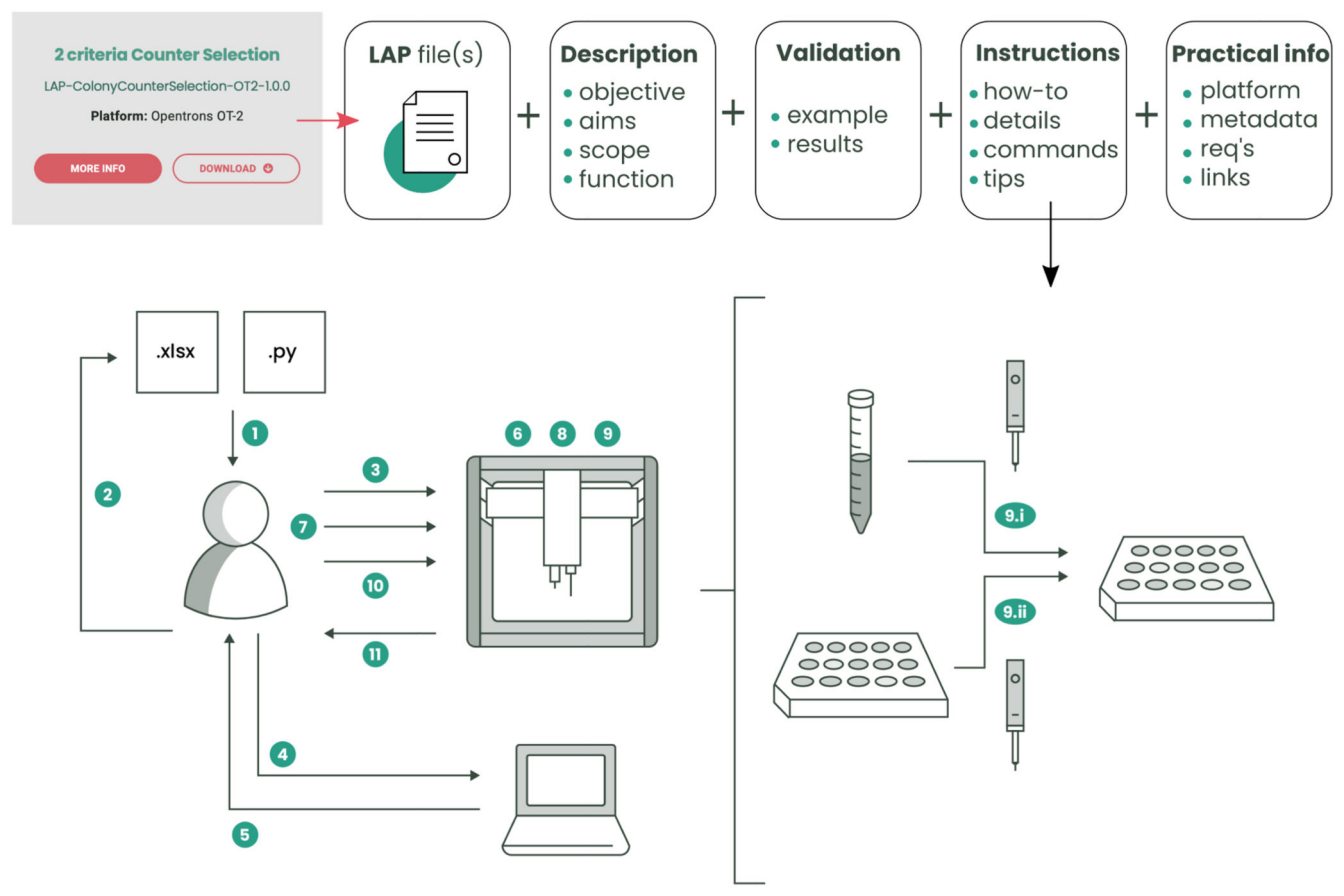
LAP information and description. LAPs can be expanded by clicking on the *more info* button. The description of each LAP follows a consistent layout, making it easy to comprehend. In addition to the LAP files, each protocol includes the following information: a high-level description of the protocol’s objectives, an example of validation that the LAP has undergone, detailed instructions on how to run the protocol, and practical information such as the platform, code versions, requirements, and other relevant details. The step-by-step instructions are accompanied by figures to enhance understanding.

## Data Availability

The LAP Repository can be accessed at the following web address: www.laprepo.com. Furthermore, all LAPs and related code are hosted in a dedicated GitHub repository, which can be found at: https://github.com/BiocomputationLab/LAPrepository.

## References

[1] Liscouski JG (1985). Laboratory automation. Journal of Chemical Information and Computer Sciences.

[2] Baker M (2016). 1,500 scientists lift the lid on reproducibility. Nature.

[3] Chapman T (2003). Lab automation and robotics: Automation on the move. Nature.

[4] Kitney R, Adeogun M, Fujishima Y, Goñi-Moreno Á, Johnson R, Maxon M, Steedman S, Ward S, Winickoff D, Philp J (2019). Enabling the advanced bioeconomy through public policy supporting biofoundries and engineering biology. Trends in biotechnology.

[5] Hallinan JS, Wipat A, Kitney R, Woods S, Taylor K, Goñi-Moreno A (2019). Future-proofing synthetic biology: educating the next generation. Engineering Biology.

[6] Amos M, Goñi-Moreno A (2018). Cellular computing and synthetic biology. Computational matter.

[7] Carbonell P, Radivojevic T, Garcia Martin H (2019). Opportunities at the intersection of synthetic biology, machine learning, and automation.

[8] Tellechea-Luzardo J, Otero-Muras I, Goñi-Moreno A, Carbonell P (2022). Fast bio-foundries: coping with the challenges of biomanufacturing. Trends in biotechnology.

[9] Alejaldre L, Anhel A-M, Goñi-Moreno A (2023). pBLAM1-x: Standardized transposon tools for high-throughput screening. Synthetic Biology.

[10] Densmore DM, Bhatia S (2014). Bio-design automation: software+ biology+ robots. Trends in biotechnology.

[11] Appleton E, Densmore D, Madsen C, Roehner N (2017). Needs and opportunities in bio-design automation: four areas for focus. Current Opinion in Chemical Biology.

[12] Goñi-Moreno A, Carcajona M, Kim J, Martinez-Garcia E, Amos M, de Lorenzo V (2016). An implementation-focused bio/algorithmic workflow for synthetic biology. ACS synthetic biology.

[13] Oliveira SM, Densmore D (2022). Hardware, Software, and Wetware Codesign Environment for Synthetic Biology. BioDesign Research.

[14] Gallup O, Ming H, Ellis T (2021). Ten future challenges for synthetic biology. Engineering Biology.

[15] Grozinger L, Amos M, Gorochowski TE, Carbonell P, Oyarzun DA, Stoof R, Fellermann H, Zuliani P, Tas H, Goñi-Moreno A (2019). Pathways to cellular supremacy in biocomputing. Nature communications.

[16] Hanson AD, Lorenzo Vd (2023). Synthetic Biology High Time to Deliver?. ACS Synthetic Biology.

[17] Wierenga R, Golas S, Ho W, Coley C, Esvelt K (2023). PyLabRobot: An Open-Source, Hardware Agnostic Interface for Liquid-Handling Robots and Accessories. bioRxiv.

[18] Bartley B, Beal J, Rogers M, Bryce D, Goldman RP, Keller B, Lee P, Biggers V, Nowak J, Weston M (2023). Building an Open Representation for Biological Protocols. ACM Journal on Emerging Technologies in Computing Systems.

[19] Madsen C, Goñi Moreno A, Palchick Z, Roehner N, Atallah C, Bartley B, Choi K, Cox RS, Gorochowski T, Griinberg R (2019). Synthetic biology open language (SBOL) version 2.3. Journal of integrative bioinformatics.

[20] Crowther M, Grozinger L, Pocock M, Taylor CP, McLaughlin JA, Mısırlı G, Bartley BA, Beal J, Goñi-Moreno A, Wipat A (2020). ShortBOL: a language for scripting designs for engineered biological systems using Synthetic Biology Open Language (SBOL). ACS synthetic biology.

[21] McLaughlin JA, Myers CJ, Zundel Z, Misirli G, Zhang M, Ofiteru ID, Goñi-Moreno A, Wipat A (2018). SynBioHub: a standards-enabled design repository for synthetic biology. ACS synthetic biology.

[22] Martónez-Garcia E, Fraile S, Algar E, Aparicio T, Velázquez E, Calles B, Tas H, Blaózquez B, Martón B, Prieto C (2023). SEVA 4.0: an update of the Standard European Vector Architecture database for advanced analysis and programming of bacterial phenotypes. Nucleic Acids Research.

[23] Hórisson J, Duigou T, Du Lac M, Bazi-Kabbaj K, Sabeti Azad M, Buldum G, Telle O, El Moubayed Y, Carbonell P, Swainston N (2022). The automated Galaxy-SynBioCAD pipeline for synthetic biology design and engineering. Nature Communications.

[24] Whitehead E, Rudolf F, Kaltenbach H-M, Stelling J (2018). Automated planning enables complex protocols on liquid-handling robots. ACS synthetic biology.

[25] Linshiz G, Stawski N, Poust S, Bi C, Keasling JD, Hillson NJ (2013). PaR-PaR laboratory automation platform. ACS synthetic biology.

[26] Bates M, Berliner AJ, Lachoff J, Jaschke PR, Groban ES (2017). Wet lab accelerator: a web-based application democratizing laboratory automation for synthetic biology. ACS synthetic biology.

[27] Tas H, Grozinger L, Goñi-Moreno A, de Lorenzo V (2021). Automated design and implementation of a NOR gate in Pseudomonas putida. Synthetic Biology.

[28] Ouyang W, Bowman RW, Wang H, Bumke KE, Collins JT, Spjuth O, Carreras-Puigvert J, Diederich B (2022). An Open-Source Modular Framework for Automated Pipetting and Imaging Applications. Advanced biology.

[29] del Olmo Lianes I, Yubero P, Gomez-Luengo A, Nogales J, Espeso DR (2023). Technical upgrade of an open-source liquid handler to support bacterial colony screening. bioRxiv.

[30] Beal J, Goñi-Moreno A, Myers C, Hecht A, de Vicente MdC, Parco M, Schmidt M, Timmis K, Baldwin G, Friedrichs S (2020). The long journey towards standards for engineering biosystems: Are the Molecular Biology and the Biotech communities ready to standardise?. EMBO reports.

[31] Beal J, Weiss R, Densmore D, Adler A, Appleton E, Babb J, Bhatia S, Davidsohn N, Haddock T, Loyall J (2012). An end-to-end workflow for engineering of biological networks from high-level specifications. ACS synthetic biology.

[32] Crowther M, Wipat A, Goñi-Moreno A (2022). A network approach to genetic circuit designs. ACS Synthetic Biology.

[33] Tas H, Goñi-Moreno A, Lorenzo Vd (2020). A standardized inverter package borne by broad host range plasmids for genetic circuit design in Gram-negative bacteria. ACS synthetic biology.

